# Clinical and genetic features of Epstein‐Barr virus‐triggered late‐onset primary hemophagocytic lymphohistiocytosis: Ten pedigrees study

**DOI:** 10.1002/ctm2.393

**Published:** 2021-05-28

**Authors:** Lili Gao, Li Yang, Liang Huang, Yi Xiao, Jinniu Deng, Miao Zheng, Hui Luo, Lijun Jiang, Min Xiao, Chunrui Li, Jianfeng Zhou

**Affiliations:** ^1^ Department of Hematology Tongji Hospital of Tongji Medical College Huazhong University of Science and Technology Wuhan Hubei China

**Keywords:** Epstein‐Barr virus (EBV), genetic features, late‐onset primary hemophagocytic lymphohistiocytosis, pedigree investigation

Dear Editor,

In this study, we reported the clinical and genetic characteristics of 10 Epstein‐Barr virus (EBV)‐triggered late‐onset primary hemophagocytic lymphohistiocytosis (HLH) patients. As HLH is a rare and devastating disorder characterized by uncontrolled immune activation resulting from the impaired function of natural killer and cytotoxic T cells.^1^ And HLH can be further classified as either primary or secondary based on the predisposing genetic deficiency.^2^, ^3^, ^4^ Although primary HLH usually arises in infants and children, the late‐onset primary HLH is increasingly reported in the literature.^5^ It has been suggested that synergetic effects of the atypical heterozygous HLH‐associated mutations and environmental triggers, including EBV infection, were associated with the pathogenesis of late‐onset primary HLH.^6^ However, the role of EBV‐infected cell subpopulation in primary HLH is unknown.

Therefore, we retrospectively investigated 10 patients with EBV‐triggered late‐onset primary HLH (occurring at over 12 years of age) from June 2013 and June 2018 in our department. According to the Diagnostic Guidelines for HLH, all of the cases met at least five of eight criteria and thereby were diagnosed as HLH, and detailed clinical parameters are listed in Table 1. Pathological studies of bone marrow, lymph node, and pleural fluids samples demonstrated that abnormal T lymphocytes, which were characterized by CD2^bri+^CD4^–^CD5^–^CD7^dim+^CD8^+^CD45RA^–^CD45RO^+^Ki67^str+^, existed in the bone marrow of case 1 and 2. In contrast, abnormal NK cell phenotype, which was marked by CD7^dim+^CD8^–^CD11b^dim/–^CD16^dim/+^CD56^dim/+/str+^CD57^–^, existed in the bone marrow for cases 7, 9, 10, and pleural effusion of case 4. No malignant clone was found in any of these patients. Despite all the patients were treated with regimens based on HLH‐2004 protocol and supportive therapy, seven of 10 patients died of disease progression or complications, and the median survival time was 3.4 months. Three patients who received allogeneic hematopoietic stem cell transplantation (allo‐HSCT) from unrelated donors acquired longer survival time compared with patients who did not receive allo‐HSCT (Figure 1A).

**TABLE 1:** Clinical and laboratory characteristics of 10 EBV‐triggered late‐onset primary HLH cases

	Case 1	Case 2	Case 3	Case 4	Case 5	Case 6	Case 7	Case 8	Case 9	Case 10	Median (range)
Gender	Female	Female	Female	Female	Female	Male	Male	Female	Male	Female	–
Age at onset (y), HLH	14	18	25	45	12	25	21	18	32	13	22.3 (12–45)
Fever (℃)	40.0	41.0	39.2	39.8	39.0	39.5	39.6	39.2	39.4	38.6	39.5 (39–41)
Neutrophils, 10^9^/L	0.3	0.2	0.8	0.1	0.6	0.1	0.7	0.9	0.6	0.8	0.5 (0.1–0.9)
Hemoglobin, g/L	64.0	88.0	88	122.0	96.0	98.0	99.0	86.0	64.0	76.0	88.1 (64–122)
Platelets, 10^9^/L	35.0	14.0	32	29.0	90.0	25.0	44.0	64.0	9.0	218.0	56.0 (14–90)
ALT index *	6.8	11.5	5.1	1.2	1.6	2.2	2.3	5.5	3.2	10.0	5.0 (1.2–11.5)
AST index **	28.0	24.0	6.2	4.2	1.5	2.2	1.2	1.8	0.9	9.3	7.7 (0.9–28)
LDH index ^#^	21.6	16.7	6.0	8.3	0.7	6.8	1.4	1.3	2.1	4.0	6.9 (0.7–21.6)
Triglycerides, mM	4.5	3.6	4.1	13.4	2.7	2.3	2.3	3.5	2.6	2.6	4.2 (2.3–13.4)
Fibrinogen, g/L	0.8	0.5	1.0	0.6	1.5	1.2	1.4	0.9	0.6	1.4	1.0 (0.5–1.5)
Ferritin, μg/L	8000	50000	3481	8000	1500	8000	5052	1228	18403	923	8787 (1228–50000)
sCD25 index^&^	6.1	6.6	2.1	7.6	4.8	2.9	8.3	3.6	5.2	4.8	5.2 (2.1–8.
Lymphadenopathy	Yes	No	Yes	Yes	No	Yes	Yes	Yes	Yes	Yes	–
Splenomegaly (thickness, cm)	5.9	4.2	5.1	6.8	2.8	3	6.7	4.3	6.4	6.5	5.2 (2.8–6.8)
Hemophagocytosis	BM	No	BM, spleen	No	BM	BM	BM	No	BM	No	–
NK‐cell activity (%)	5.6 ± 0.6	8.9± 0.7	1.2 ± 0.3	7.5 ± 1.3	4.0 ± 0.5	16.8 ± 2.6	16.0 ± 3.1	4.6 ± 0.8	15.8 ± 3.2	4.3 ± 0.7	–
Perforin (%)	61.2 ± 4.7	50.8 ± 3.8	0.9 ± 0.2	27.2 ± 2.6	31.2 ± 2.4	76.6 ± 5.4	11.9 ± 1.2	52.3 ± 5.0	17.6 ± 1.8	26.6 ± 3.8	–
Degranulation of resting NK cells (%)	1.1 ± 0.2	6.7 ± 0.8	12.6± 1.8	2.3 ± 0.6	13.2 ± 1.6	1.3 ± 0.3	14.5 ± 3.0	27.7 ± 4.7	17.2 ± 1.7	0.4 ± 0.1	–
Degranulation of IL‐2 stimulated NK cells (%)	1.4 ± 0.1	42.8 ± 2.7	42.1 ± 3.6	3.1 ± 0.2	67.9 ±5.7	2.8 ± 0.3	72.4 ± 6.6	49.3 ± 3.9	56.9 ± 4.6	73.1± 6.3	–
Sorting‐PCR	NK	NK	NK	NK	NK and T	NK	NK	NK	NK	NK	–
Sorting‐FISH	ND	ND	ND	ND	ND	ND	ND	NK	ND	NK	–
Treatment	HLH‐2004	HLH‐2004	HLH‐2004	HLH‐2004; MDL	HLH‐2004; BR	HLH‐2004; P‐Gemox; Allo‐HSCT	RCHOP; HLH‐2004; P‐Gemox	HLH‐2004; Mp; Allo‐HSCT	HLH‐2004	HLH‐2004 Allo‐HSCT	–
Disease reactivation	Yes	Yes	Yes	Yes	Yes	Yes	Yes	Yes	Yes	Yes	–
Outcome	Death	Death	Death	Death	Death	Alive	Death	Alive	Death	Alive	–
Cause of death	ICH	PD	PD	PD	Respiratory failure	–	PD	–	PD	–	–
Survival time (days)	35	27	112	95	199	906	180	907	66	575	–

Abbreviations: Allo‐HSCT, allogeneic hematopoietic stem cell transplantation; BM, bone marrow; BR, brentuximab vedotin and rituximab; EBV, Epstein‐Barr virus; HLH, hemophagocytic lymphohistiocytosis; MDL, methotrexate, dexamethasone, and L‐asparaginase; ICH, intracerebral hemorrhage; Mp, methylprednisolone; ND, not done; PD, progressive disease; P‐Gemox, pegaspargase, gemcitabine, and oxaliplatin; RCHOP, rituximab, cyclophosphamide, doxorubicin, vincristine, and prednisone; sCD25: soluble CD25; sorting‐FISH: immunobead sorting followed by fluorescence in situ hybridization; sorting‐PCR, immunobead sorting followed by quantitative PCR; y: year.

* ALT index, patient's ALT/upper normal limit of ALT;

** AST index, patient's AST/upper normal limit of AST;

^#^ LDH, patient's LDH/upper normal limit of LDH;

^&^ sCD25 index, patient's sCD25/upper normal limit of sCD25.

**FIGURE 1:**
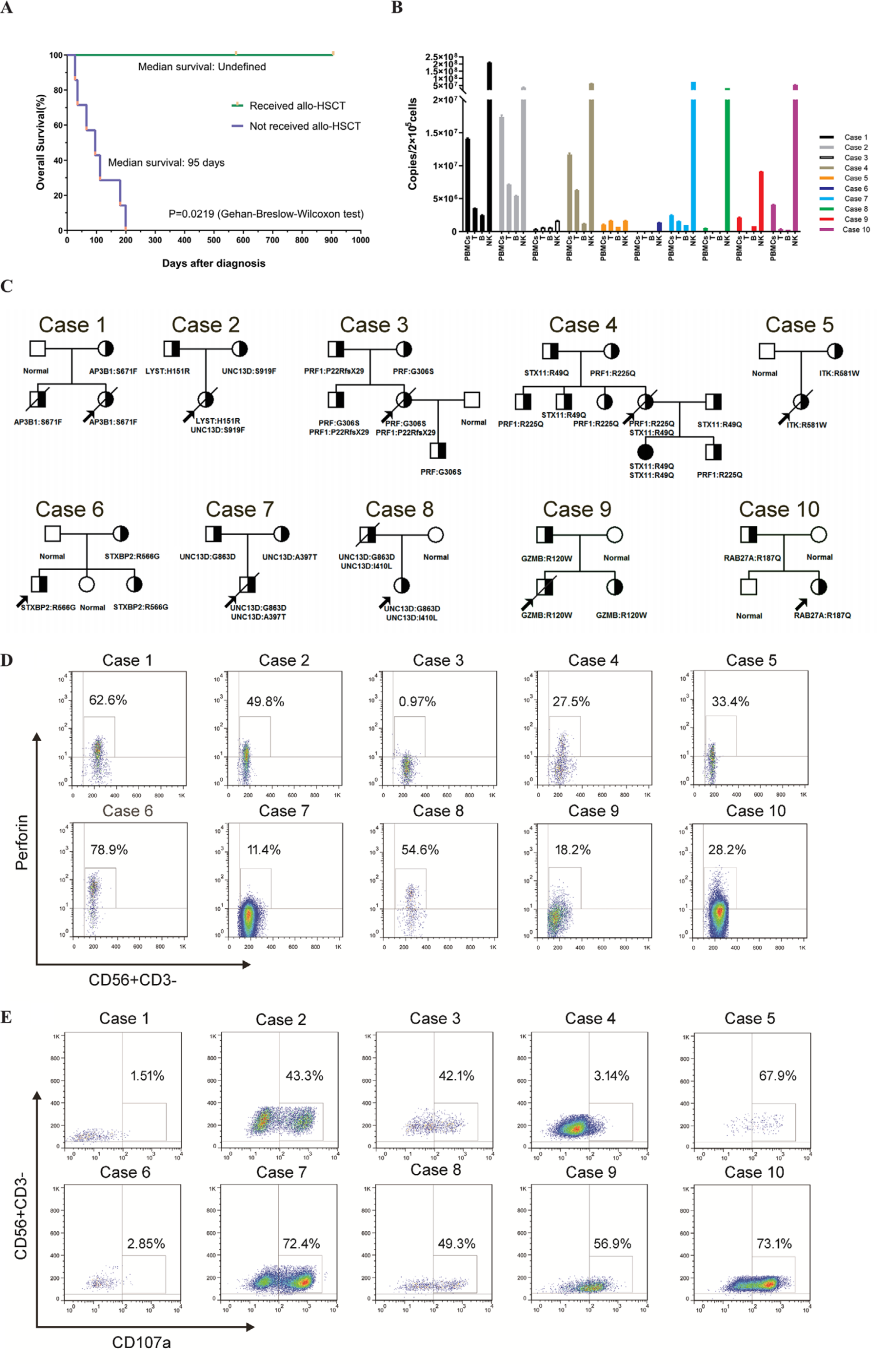
(A) Kaplan‐Meier survival curves of late‐onset primary HLH patients who received or not received allo‐HSCT. *p* values calculated by the Gehan‐Breslow‐Wilcoxon test. (B) Target cell identification by sorting‐PCR in the late‐onset primary HLH patients. EBV‐DNA copies quantification of different cell types (PBMCs, T, B, and NK) by qualitative PCR demonstrated that EBV primarily infected NK cells in 100% (10/10) of them, and concomitant infection in T cells in 10% of patients (1/10). (C) The pedigrees of the 10 families affected by hemophagocytic lymphohistiocytosis were included in the present study. Squares, males; circles, females; slash, deceased; half‐filled, heterozygous; darkened, homozygous; arrows, probands of the families. The affected genes and amino acid substitution caused by mutations in each family are indicated below the corresponding pedigree. (D) The expression of perforin protein on NK cells of case 2, case3, case 4, case 5, case 7, case 9, and case 10 was decreased, while relative regular perforin expression on CD56^+^CD3^–^NK cells was observed in case 1, case 6, and case 8. Numbers indicate the percentage of perforin expression. (**E)** The degranulation of rIL‐2 stimulated NK cells (CD56^+^CD3^–^) was determined by the expression of CD107a. The results showed that stimulated degranulation of NK cells was significantly decreased in case 1, case 4, and case 6; however, relatively healthy stimulated degranulation of NK cells was observed in case 5, case 7, case 9, and case 10. Numbers indicate the percentage of CD107a expression Abbreviation: PBMCs, peripheral blood mononuclear cells.

Firstly, to investigate the cell subpopulation pattern of EBV infection, immunobead sorting followed by quantitative PCR (sorting‐PCR) and fluorescence in situ hybridization (sorting‐FISH) assay was performed. Peripheral blood mononuclear cells (PBMCs) were isolated and fractionated into CD3^+^, CD19^+^, and CD56^+^ cells using an immunobead method, purities of which were confirmed by flow cytometry to be 97%–99% for B and T cells and 91%–95% for NK cells.^7^ Then purified cells were analyzed by quantitative PCR assay and fluorescence in situ hybridization with the EBER probe (green) as previously described.^7^ The results demonstrated that T cells, B cells, and NK cells were all shown to be infected with EBV, while EBV significantly infected NK cells in nine patients (cases 1–4, 6–10). EBV predominantly infected both NK cells and T cells in one patient (case 5) (Figure 1B). Sorting‐FISH was performed in two cases (cases 8 and 10). EBV was found predominantly in NK cells, consistent with the results of sorting‐PCR.

Secondly, to understand the pathogenesis of EBV‐triggered late‐onset primary HLH, we carried out next‐generation sequencing (NGS) using a custom design for the HLH panel under the Ion AmpliSeq Ready‐to‐Use custom designer platform following the guide (https://www.ampliseq.com/protected/dashboard.action), and detailed NGS procedure has been reported in previous literature.^8^ As described in Table 2, a total of five cases had one single gene mutation at heterozygous status (cases 1, 5, 6, 9, and 10), two cases had gene mutations in two primary HLH‐related genes at heterozygous state (cases 2 and 4). Case 3 had a compound heterozygous mutation in PRF1 (this case had been reported in the previous article^8^), case 7 had a compound heterozygous mutation in UNC13D, while case 8 had two sites of heterozygous mutations in UNC13D, which were descended from her father. Most of the mutations were predicted to be damaging by software SIFT (Sorting Intolerant From Tolerant). All the details of 10 pedigrees are shown in Figure 1C and Table 2.

**TABLE 2:** The EBV‐DNA load and genetic characteristics of 10 late‐onset primary HLH cases and their families

Case number	Relationship	Age (y)	EBV (copies/μg)	Gene	Protein	1000 genome	SIFT (<0.05)
Case 1	Proband	14	(1.2 ±0.1) × 10^7^	AP3B1	S671F	–	0.049 (Damaging)
	Father	40	<5 × 10^2^	Normal	–		
	Mother	39	<5 × 10^2^	AP3B1	S671F		
	Brother	0.75	<5 × 10^2^	AP3B1	S671F		
Case 2	Proband	18	(3.3 ±0.5) × 10^7^	LYST	H151R	–	0.042 (Damaging)
				UNC13D	S919F	–	0.699 (Tolerated)
	Father	50	<5 × 10^2^	LYST	H151R		
	Mother	49	<5 × 10^2^	UNC13D	S919F		
Case 3	Proband	25	(5.4 ± 1.5) × 10^5^	PRF1	G306S	–	0.010 (Damaging)
				PRF1	P22RfsX29	–	–
	Father	48	(4.1 ±1.1) × 10^3^	PRF1	P22RfsX29		
	Mother	47	<5 × 10^2^	PRF1	G306S		
	Brother	23	<5 × 10^2^	PRF1	G306S		
				PRF1	P22RfsX29		
	Son	1	(3.2 ±0.8) × 10^3^	PRF1	G306S		
Case 4	Proband	45	(1.9±0.3) × 10^7^	STX11	R49Q	0.02	1.000 (Tolerated)
				PRF1	R225Q	–	0.366 (Tolerated)
	Father	70	<5 × 10^2^	STX11	R49Q		
	Mother	69	<5 × 10^2^	PRF1	R225Q		
	Brother	47	<5 × 10^2^	STX11	R49Q		
	Brother	43	<5 × 10^2^	PRF1	R225Q		
	Sister	40	<5 × 10^2^	PRF1	R225Q		
	Son	16	<5 × 10^2^	PRF1	R225Q		
	Daughter	18	<5 × 10^2^	STX11(hom)	R49Q		
	Husband	48	<5 × 10^2^	STX11	R49Q		
Case 5	Proband	12	(1.3 ±0.4) × 10^7^	ITK	R581W	0.001	0.007 (Damaging)
	Father	39	<5 × 10^2^	Normal			
	Mother	37	<5 × 10^2^	ITK	R581W		
Case 6	Proband	25	(7.7 ±1.9) × 10^5^	STXBP2	R566G	0.012	0.035 (Damaging)
	Father	50	<5 × 10^2^	Normal			
	Mother	49	<5 × 10^2^	STXBP2	R566G		
	Elder Sister	27	<5 × 10^2^	STXBP2	R566G		
	Younger Sister	23	<5 × 10^2^	Normal			
Case 7	Proband	21	(1.3±0.7) × 10^7^	UNC13D UNC13D	G863D A397T	0.001 ‐	0.000 (Damaging) 0.262 (Tolerated)
	Father	45	<5 × 10^2^	UNC13D	G863D		
	Mother	44	<5 × 10^2^	UNC13D	A397T		
Case 8	Proband	18	(6.5±1.3) × 10^7^	UNC13D UNC13D	G863D I410L	0.001 0.003	0.000 (Damaging) 1.000 (Tolerated)
	Father	43	<5 × 10^2^	UNC13D UNC13D	G863D I410L		
	Mother	42	<5 × 10^2^	Normal			
Case 9	Proband	32	(4.0 ± 1.1) × 10^6^	GZMB	R120W	–	0.031 (Damaging)
	Father	54	(1.2 ±0.3) × 10^3^	GZMB	R120W		
	Mother	53	<5 × 10^2^	Normal	–		
	Sister	35	<5 × 10^2^	GZMB	R120W		
Case 10	Proband	13	(1.2±0.4) × 10^7^	RAB27A	R187Q	0.009	0.372 (Tolerated)
	Father	38	<5 × 10^2^	RAB27A	R187Q		
	Mother	36	(1.2±0.6) × 10^4^	Normal	–		
	Brother	8	<5× 10^2^	Normal	–		

1000 genome, also known as 1000 genome project, is a map of human genome variation from population‐scale sequencing, the number stands for the frequency of amino acid substitution in the database. "‐" means amino acid substitution can't be detected in the database. SIFT predicts whether an amino acid substitution affects protein function. The lower the score, the more likely to be harmful.

Abbreviations: hom, homozygote; y, year.

Thirdly, to evaluate the impact of genetic mutations on their NK cell function, NK‐cell activity assay, NK‐cell degranulation assay, and perforin staining assay were subsequently performed on whole blood samples from these pedigrees. NK‐cell activity assay was performed as previously described.^8^ Perforin staining assay was performed by staining PBMC samples for NK cell markers before fixation and permeabilization. Then anti‐perforin or phycoerythrin‐conjugated mouse immunoglobulin G2b (BD Pharmingen) was stained. NK cells were first gated and subsequently analyzed for the expression of the PRF1 protein. As for the NK‐degranulation assay, PBMCs were incubated with or without rIL‐2 to investigate activated or resting NK‐cell degranulation, respectively. For analysis, lymphocytes were gated based on forward scatter, then CD3^–^CD56^+^ NK cells were gated and assessed for surface expression of CD107a. Results demonstrated that reduced NK‐cell activity was observed in all EBV‐triggered late‐onset primary HLH patients (Table 1), while NK‐cell activity defect was found in four patients, which was consistent with previous research.^9^ Substantially reduced perforin expression on CD3^–^CD56^+^ NK cells was detected in seven patients (Figure 1D). One case had abnormal resting NK‐cell degranulation, and four cases had defective resting NK‐cell degranulation. Abnormal activated NK‐cell degranulation was recorded in three cases (Figure 1E). Nine cases had either reduced perforin expression or decreased activated NK‐cell degranulation.

Based on the above findings, our study demonstrated that, unlike sporadic HLH, EBV‐triggered late‐onset primary HLH usually has one or two pathologic mutations of primary HLH‐associated genes, reduced perforin expression, NK activity, or degranulation. Most patients have high load EBV infection, and the target cells of EBV are usually NK cells, sometimes with T cells, which was typical for FHL (familial hemophagocytic lymphohistiocytosis) patients.^10^ Through the pedigree investigation, we also noticed that although some family members of the patient had the same mutations, they did not develop the disease, possibly attributing to their lack of EBV infection or other "second‐hit" factors such as additional genetic mutations. Therefore, genetic mutations, as well as EBV‐infected lymphocyte subtypes, may collectively be involved in the pathogenesis of late‐onset primary HLH. HLH gene sequencing and pedigree investigation, combined with EBV‐infected cell type identification, are valuable in the differential diagnosis of late‐onset primary HLH. Timely allo‐HSCT is recommended to improve the poor prognosis of late‐onset primary HLH.

## CONFLICT OF INTEREST

The authors declare that they have no conflict of interest.

## AUTHOR CONTRIBUTIONS

All authors contributed to the study conception and design. Material preparation, data collection, and analysis were performed by Liang Huang, Yi Xiao, Jinniu Deng, Miao Zheng, Hui Luo, and Lijun Jiang. The first draft of the manuscript was written by Lili Gao with the help of Li Yang and revised by Chunrui Li, Min Xiao, and Jianfeng Zhou. All authors commented on previous versions of the manuscript and agreed on the final manuscript.
